# Human Parvovirus 4 Infection among Mothers and Children in South Africa

**DOI:** 10.3201/eid2104.141545

**Published:** 2015-04

**Authors:** Philippa C. Matthews, Colin P. Sharp, Amna Malik, William F. Gregory, Emily Adland, Pieter Jooste, Philip J. R. Goulder, Peter Simmonds, Paul Klenerman

**Affiliations:** University of Oxford, Oxford, UK (P.C. Matthews, A. Malik, E. Adland, P.J.R. Goulder, P. Klenerman);; Oxford University Hospitals, Oxford (P.C. Matthews, P. Klenerman);; The University of Edinburgh, Midlothian, Scotland, UK (C.P. Sharp, W.F. Gregory, P. Simmonds);; University of Free State, Kimberley, South Africa (P. Jooste);; NIHR Biomedical Research Centre, Oxford (P. Klenerman)

**Keywords:** parvovirus, PARV4, HIV, South Africa, prevalence, co-infection, serology, human, viruses

**To the Editor:** Human parvovirus 4 (PARV4) is a single-stranded DNA virus in the family *Parvoviridae* (*1*). In Western countries, IgG against PARV4 is largely found only in persons with risk factors for parenteral infection and is strongly associated with co-infection with bloodborne viruses (*2*–*4*). In Africa, transmission seems to be more complicated; reported PARV4 seroprevalence is 4%–37%, even among persons at low risk and with no evidence of HIV or hepatitis C virus (HCV) co-infection (*1*,*5*,*6*).

The clinical significance of PARV4 infection remains uncertain. Infections may be asymptomatic, but a variety of clinical associations have been reported (*1*,*7*), including an increased rate of progression to AIDS in persons co-infected with HIV (*8*). This association raises particular concerns for many African populations in which these viruses are co-endemic.

To characterize the epidemiology of PARV4 infection in South Africa, we studied adults and children from pediatric outpatient clinics in Kimberley, South Africa, during May 2009–August 2013. Of the 157 participants, 90 were HIV-1–infected children, 24 their HIV-negative siblings, and 43 HIV-1–infected mothers (of whom 4 had >1 child enrolled). Approval was given by the Ethics Committee of the Faculty of Health Science, University of Free State, South Africa. Written consent was given by all adults and parents/guardians on behalf of their children.

Blood samples were collected from participants, and serum was tested for evidence of PARV4 infection by using ELISA (in duplicate) to detect IgG against PARV4 viral protein 2 (*3*,*6*) and by using PCR to detect PARV4 DNA (*9*). For 92 patients, HIV RNA loads were available; testing was performed by using the Abbott Laboratories m2000 platform (Abbott Park, IL, USA). For 118 of the HIV-infected patients, CD4+ T-cell counts were ascertained by flow cytometry. Statistical analyses were undertaken by using Prism version 6.0f and online software (http://graphpad.com/quickcalcs/). Confidence intervals were calculated by using the adjusted Wald method (http://www.measuringusability.com/wald.htm).

We detected IgG against PARV4 in 58 (37%) of 157 patients; this proportion is broadly comparable with that reported from other settings in sub-Saharan Africa, including Burkina Faso, the Democratic Republic of the Congo, and a previous cohort of HIV-infected persons in South Africa (*5*). Although routes of transmission in Africa remain to be characterized, these high seroprevalence rates support the possibility that some PARV4 transmission may be occurring by nonparenteral routes, as suggested by others (*5*,*10*).

PARV4 IgG seroprevalence was higher among adults (49%) than children (33%), although this difference did not reach statistical significance (p = 0.07, Fisher exact test; Figure, panel A). We found a significant relationship between increasing age and PARV4 IgG serostatus (R^2^ = 0.59 by linear regression, p = 0.025; Figure, panel B). The numbers in each group are small, and further work is needed to define this association with more confidence. We did not detect any cases of PARV4 viremia, suggesting that chronic viremia or reactivation are probably uncommon, even among HIV-infected patients.

**Figure:**
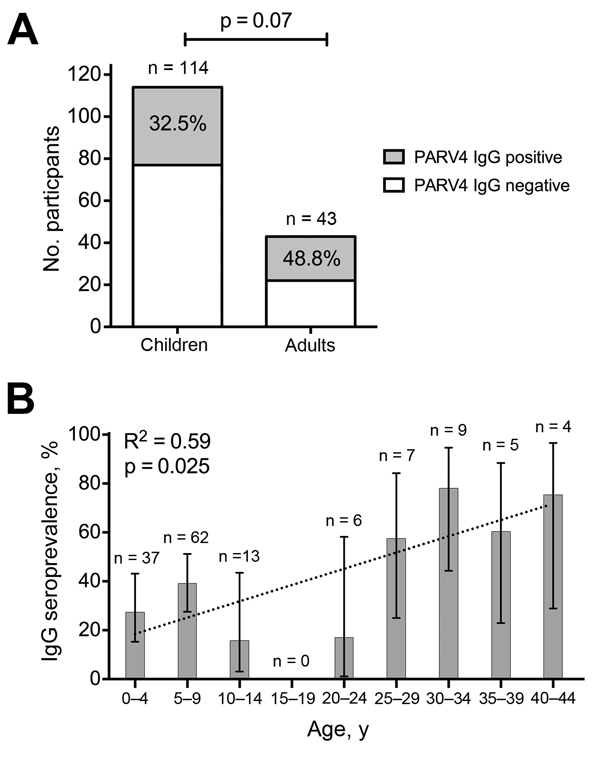
Relationship between age and seroprevalence of IgG against human parvovirus 4 (PARV4) among 157 mothers and children in Kimberley, South Africa, 2009–2013. A) Number and proportion of children and adults seropositive for IgG against PARV4; the number in each group is shown above the bar. p value calculated by using the Fisher exact test. B) Proportion of population seropositive for IgG against PARV4 according to age; the number in each group is shown above the bar. Data are shown for 143 persons because no date of birth was recorded for 2 children and 12 adults. Error bars show 95% CIs calculated by the adjusted Wald method. R^2^ was calculated by linear regression (dotted line). We considered whether maternal antibodies might be contributing to PARV4 IgG seroprevalence among those 0–4 years of age. However, from 11 children in this group who were <12 months of age (in whom detection of maternal antibody might still be expected), 2 were PARV4 IgG seropositive, and only 1 of these had an IgG-positive mother, suggesting that maternal antibodies did not contribute significantly to PARV4 seropositivity in this cohort.

On the basis of previously reported data demonstrating PARV4 viremia in neonates (*7*), we hypothesized that vertical transmission is possible. To investigate further, we sought evidence of concordance between IgG serostatus of mothers and their children. Maternal PARV4 IgG status did not differ between IgG-positive and IgG-negative children (p = 1.00, Fisher exact test; Technical Appendix Table 1). The absence of correlation between the IgG statuses of mothers and children suggests that vertical transmission is probably not a major contributor to new infections, although it remains plausible that it may sometimes occur.

Data from Europe that suggest an association between PARV4 infection and progression to Centers for Disease Control and Prevention B-syndromes in HIV-positive persons are problematic because of confounding high rates of HCV infection and injection drug use in the PARV4-positive group (*8*). We sought evidence for this effect in our cohort, in which rates of HCV infection and injection drug use were likely to be negligible. We found no evidence of a PARV4 serostatus effect on HIV RNA load or CD4+ T cells in children (p = 0.13, p = 0.68, respectively; Technical Appendix Table 1) or adults (p = 0.15, p = 0.77, respectively; Technical Appendix Table 2).

We found an unexpected negative correlation between PARV4 IgG and HIV status in children (p = 0.05, Fisher exact test; Technical Appendix Table 1). One possible explanation is that a detectable PARV4 IgG response is not mounted or maintained in the context of HIV infection; however, this theory is not supported by previous studies in which PARV4 IgG seems to be more prevalent in HIV-infected populations (*5*,*8*).

Our analysis was limited by small numbers tested and the retrospective approach to sample testing. Demographic data were not recorded for this cohort, so we are unable to explore further possible social or demographic risk factors that might correlate with PARV4 infection.

This study contributes to an evolving body of data suggesting that PARV4 is highly endemic to different settings across Africa. The unknown clinical effects and transmission routes of this virus remain pressing questions for future research.

Technical Appendix Characteristics of 114 children and 43 HIV-positive adults according to parvovirus 4 IgG serostatus, Kimberley, South Africa, 2009–2013.
