# Supplementary material for: Human Parvovirus 4 Infection among Mothers and Children in South Africa
Source: Emerg Infect Dis. 2015 Apr;21(4):713–5. doi: 10.3201/eid2104.141545 (PMC4378500; doi:10.3201/eid2104.141545)
Supplement: Technical Appendix — Characteristics of 114 children and 43 HIV-positive adults according to parvovirus 4 IgG serostatus, Kimberley, South Africa, 2009–2013. [file 14-1545-Techapp-s1.pdf]

Article DOI: http://dx.doi.org/10.3201/eid2104.141545

Human Parvovirus 4 Infection among Mothers and Children in South Africa

Technical Appendix

Technical Appendix Table 1. Characteristics of 114 children in Kimberley, South Africa, according to PARV4 IgG serostatus*

Characteristic	Parv4 IgG negative, n = 77	Parv4 IGg positive, n = 37	p-value
Median age (IQR), y	7 (3–9)	7 (4–8)	p = 0.87†
Number of males (%)	38 (49)	12 (32)	p = 0.21‡
Number with HIV (%)	65 (85)	25 (68)	p = 0.05‡
Median CD4+ T cell percentage (IQR)§	25 (18-30)	24 (16-30)	p = 0.68‡
Median HIV viral load (IQR), copies/mL§	120,000 (21,600–365,000)	52,000 (7,700–150,000)	p = 0.13†
Proportion with PARV4 IgG+ mother (%)¶	15/33 (45)	7/14 (50)	p = 1.00‡

*IQR, interquartile range; PARV4, parvovirus 4.
†Mann-Whitney U test.
‡Fisher exact test.
§CD4+ T cells and HIV viral loads are reported for HIV-infected persons only.
¶47 mother–child pairs represented (from a total of 43 mothers recruited; 4 have 2 children each. Numerators in this row add up to 22 because 1 PARV4-positive mother had 2 children).

Technical Appendix Table 2. Characteristics of 43 HIV-positive adults in Kimberley, South Africa, according to PARV4 IgG serostatus*

Characteristic	PARV4 IgG negative, n = 22	PARV4 IgG positive, n = 21	p-value†
Median age (IQR), y	26 (22–35)	33 (28–39)	p = 0.09
Median CD4+ T cell count (IQR), cells/mm^3	318 (261–440)	322 (221–469)	p = 0.77
Median HIV RNA Load (IQR), copies/mL	23,199 (5,050–89,930)	93,000 (8,400–207,500)	p = 0.15

*IQR, interquartile range; PARV4, parvovirus 4.
†Mann-Whitney U test.
